# Toward Drift-Aware Multimodal Early Warning for Acute Exacerbations of Chronic Obstructive Pulmonary Disease

**DOI:** 10.2196/98345

**Published:** 2026-08-10

**Authors:** Haoran Li, Shuaihua Fan, Xiaojuan Li, Nina Liu, Huaiqing Qi, Jun Guo

**Affiliations:** 1 Department of Respiratory and Critical Care Medicine, Beijing Tsinghua Changgung Hospital, School of Clinical Medicine, Tsinghua Medicine, Tsinghua University, 168 Litang Road, Changping District, Beijing, China, +86 13681573493; 2 Yichang Central People's Hospital, The First College of Clinical Medical Science, China Three Gorges University, Yichang, Hubei, China; 3 Tsinghua University, Beijing, China

**Keywords:** chronic obstructive pulmonary disease, COPD, acute exacerbation, environmental exposure, early warning system, concept drift

## Abstract

The clinical management of acute exacerbations of chronic obstructive pulmonary disease (AECOPD) is increasingly moving from reactive treatment to proactive early warning. However, existing environmental forecasting approaches remain constrained by heterogeneous exposure-response relationships, limited AECOPD-specific evidence for digital behavioral surveillance, and vulnerability to concept drift under nonstationary social and health care conditions. This viewpoint argues for a drift-aware multimodal early warning research agenda that integrates environmental exposure data, candidate digital behavioral signals, and routinely collected clinical burden indicators. Drawing on representative literature from environmental epidemiology, respiratory medicine, digital epidemiology, and medical informatics, we discuss current methodological challenges and potential directions for future AECOPD surveillance. Particular attention is paid to multimodal data integration, concept drift, adaptive state-space modeling, regime-aware handling of structural breaks, and equity-related challenges associated with digital behavioral data. Rather than presenting a validated forecasting system, we outline conceptual design considerations for future AECOPD-specific studies, including multimodal data fusion, state-space adaptive updating, drift subtype diagnosis, and subgroup-aware validation strategies. We also highlight important evidence gaps, particularly regarding the use of internet search queries and social media signals for AECOPD prediction. Drift-aware multimodal surveillance represents a promising direction for future AECOPD early warning, but substantial methodological, clinical, and implementation challenges remain. Future research should prioritize disease-specific validation, transparent evaluation of adaptive forecasting methods, and equitable deployment across populations with differing levels of digital access and health care resources.

## Introduction

Chronic obstructive pulmonary disease (COPD) is a leading cause of morbidity and mortality worldwide, and imposes a substantial burden on health care systems. Its burden is unevenly distributed, with higher risks among older adults; rural residents; and socioeconomically disadvantaged populations, particularly in low- and middle-income settings [1,2]. In China, the absolute number of COPD cases and disability-adjusted life years remains exceptionally high, creating sustained pressure on clinical and public health resources [3]. Conventional COPD management remains largely reactive, focusing on treatment after acute exacerbations have already occurred. Because acute exacerbations of COPD (AECOPD) worsen patient outcomes and increase direct and indirect health care costs, there is a need to move toward proactive early warning based on high-risk environmental triggers, candidate digital behavioral signals, and routinely collected clinical burden indicators [4,5]. In this viewpoint, “multimodal” refers to the integration of 3 operational data streams: environmental exposure data, digital behavioral signals, and clinical burden data. Molecular biomarkers are discussed only as future patient-level extensions, not as part of the core real-time early warning architecture.

## Evidence Base and Scope of This Viewpoint

This viewpoint is not intended as a systematic review or quantitative evidence synthesis. Instead, it draws on representative literature from environmental epidemiology, respiratory medicine, digital epidemiology, and medical informatics to develop a conceptual research agenda for drift-aware multimodal early warning of AECOPD. We prioritized studies that informed one or more of the following components: environmental exposure-response relationships, digital behavioral surveillance, nonstationary time-series forecasting, concept drift, and adaptive state-space modeling. Molecular and cellular studies were considered only when they helped establish biological plausibility for environmental triggers rather than as direct inputs to the proposed forecasting architecture. Therefore, the evidence base should be interpreted as illustrative and hypothesis generating rather than exhaustive.

## Dual Exposures to Meteorological Factors and Air Pollution: Limitations and Advances in Epidemiological Research

### Environmental Exposure Signals and Biological Plausibility

Environmental exposures are central candidate inputs for AECOPD early warning because they are externally measurable, temporally dynamic, and biologically plausible triggers of respiratory deterioration. Among meteorological variables, low temperature and temperature variability have been repeatedly associated with increased COPD morbidity and mortality. A recent systematic review and meta-analysis reported a nonlinear association between extreme temperature exposure and COPD morbimortality, with cold exposure serving as an important independent risk factor [6]. Importantly, cold-related AECOPD risk is not limited to same-day exposure. There is evidence from the city of Panzhihua, China, suggesting that the morbidity risk associated with low temperature may peak approximately 2 days after exposure and that cumulative effects can persist for up to 16 to 17 days [7]. Similar time-series evidence from Hangzhou, China, further supports the association between short-term extreme temperature exposure and increased mortality risk among patients with COPD [8]. From a forecasting perspective, these findings support the inclusion of temperature as a lagged environmental covariate, distributed-lag exposure term, rolling exposure summary, or time-varying coefficient rather than as a static background risk factor.

Ambient air pollution represents a second major external exposure domain for AECOPD surveillance. This emphasis is consistent with the Global Initiative for Chronic Obstructive Lung Disease 2023 committee report, which recognizes air pollution as a clinically relevant COPD exposure domain and supports the need to incorporate air quality information into respiratory risk assessment [5]. Epidemiological studies have linked air pollution exposure with impaired pulmonary function, increased COPD risk, and adverse COPD outcomes [9]. Specifically, particulate matter exposure, including PM_2.5_ and PM_10_, has been associated with increased COPD mortality, with evidence that lag effects and population vulnerability may vary across geographic regions and population groups [10]. These findings suggest that air pollution variables should be treated as dynamic covariates whose effects may vary by season, concurrent meteorological conditions, baseline population vulnerability, and local exposure patterns. Therefore, PM_2.5_, PM_10_, and related pollutants may be incorporated into early warning models alongside temperature, humidity, and other meteorological variables to capture time-varying environmental risk.

Molecular and cellular studies provide biological plausibility for these macroepidemiological associations, but they should not be interpreted as direct evidence that molecular pathways can currently be used as routine real-time forecasting inputs. Cold exposure may contribute to airway dysfunction through impaired mucociliary clearance, airway hyperresponsiveness, mucus dysregulation, and inflammatory activation involving temperature-sensitive airway mechanisms such as TRPM8 signaling [11-14]. Similarly, PM_2.5_ exposure may promote oxidative stress, endothelial injury, mitochondrial dysfunction, and inflammatory responses through pathways involving redox imbalance, epigenetic regulation, and mitophagy-related injury [15-17]. However, these molecular mechanisms are not yet suitable as core inputs for most population-level AECOPD early warning systems because routine clinical and public health infrastructures generally lack standardized, low-cost, and high-frequency biomarker collection. In the conceptual architecture discussed in this viewpoint, molecular evidence is therefore used primarily to support biological plausibility for environmental exposure signals. Future personalized extensions may evaluate whether selected biomarkers can serve as patient-level covariates, mechanistic priors, or stratification variables within state-space or other adaptive forecasting models.

### Methodological Bottlenecks in Assessing Combined Environmental Exposures

Notably, COPD risk factors in real-world settings exhibit distinct combined exposure patterns. Although the interactions between ambient temperature and air pollution in disease progression have been preliminarily identified, the current research framework lacks standardized metrics for assessing combined exposure. Recent high-resolution time-series analyses confirm that the health consequences of extreme environmental exposures are far from a simple linear superposition of single variables. For instance, extreme ambient temperature and air pollution exhibit a significant positive synergistic effect in driving AECOPD hospitalization and mortality burdens [18,19]. Furthermore, authentic exposure scenarios typically involve a “mixture” of pollutants. Empirical evidence indicates that, beyond the dual impact of temperature and PM_2.5_, the introduction of humidity further amplifies the respiratory toxicity of PM_2.5_ [20] and long-term exposure to a mixture of air pollutants exerts a driving force on COPD progression that substantially exceeds the independent effects of single pollutants [21]. This multidimensional synergistic stress also demonstrates profound demographic and spatial heterogeneity: short-term exposure to air pollutants poses a particularly severe mortality risk to older adult populations [22], and intrinsic differences exist between urban and rural areas regarding the lagged patterns of particulate matter–induced COPD mortality [10]. Concurrently, household air pollution or combined ozone exposure plays a pivotal role in inducing COPD among specific demographic groups (eg, younger adults) and in specific high-altitude regions [23,24]. Established environmental mixture methods may help address part of this bottleneck. Weighted quantile sum regression, quantile g-computation, and Bayesian kernel machine regression have been used to evaluate joint pollutant effects, identify influential components within exposure mixtures, and allow for potential nonlinear or nonadditive associations. Although these methods are primarily designed for exposure-response estimation rather than real-time forecasting, they may inform the construction of mixture-derived or interaction-aware exposure features for future AECOPD early warning models. Therefore, future early warning studies should consider composite or interaction-aware exposure representations that can capture cold-pollution coexposure without assuming simple additive effects [19].

## Paradigm Evolution of AECOPD Early Warning Models

### Transition From Static Statistical Analysis to Dynamic Time-Series Modeling

Historically, risk assessment for AECOPD has relied heavily on peripheral blood inflammatory biomarkers (eg, neutrophil-to-lymphocyte ratio and monocyte-to-lymphocyte ratio) and traditional cross-sectional explanatory statistical models [25]. At the level of individual prognostic assessment, conventional risk scoring systems based on baseline clinical characteristics—such as the CORE score, which is designed to predict the 1-year readmission risk of patients with COPD—provide quantitative tools for personalized discharge management [26]. However, these static clinical models focus primarily on intrinsic physiological vulnerability. Whether based on biomarkers or static risk scores, these approaches essentially provide a retrospective summary or lagged mapping of previous pathological damage, lacking the capacity for prospective population-level early warning against high-risk macroenvironmental triggers. With the expanding dimensionality of environmental and meteorological data, the AECOPD early warning framework is undergoing a paradigm evolution from clinical explanatory statistics to environmental predictive time-series modeling. However, conventional multivariable regression models often fail to disentangle the seasonal cycles and lagged cumulative effects inherent in environmental exposure data. To address these limitations, distributed-lag nonlinear models (DLNMs) and seasonal autoregressive integrated moving average (SARIMA) have been widely adopted within respiratory disease prediction frameworks. These time-series models demonstrate superior fitting performance, particularly for large-scale, cross-regional macrodata. For instance, a comprehensive time-series analysis of 10 major regions in Brazil successfully used DLNMs to quantify the complex exposure-response relationship between mean daily temperature and COPD mortality, validating the independent role of abnormal temperature exposure as a driver of elevated mortality risk [27]. Due to its capacity to capture long-term trends and seasonal fluctuations in time-series data, the SARIMA model was used by a Canadian research group to forecast hospitalization and emergency department (ED) visit loads for patients with COPD in specific regions. By quantifying future health care service demands, the aforementioned study provided a prospective basis for decision-making among clinicians, health care administrators, and health policymakers, thereby addressing increasing resource allocation challenges [28].

### Contemporary Forecasting Alternatives and the Role of Adaptive State-Space Thinking

The methodological landscape of health time-series forecasting has expanded beyond DLNMs and SARIMA. DLNMs remain particularly useful for estimating nonlinear and lagged exposure-response associations, making them valuable for environmental epidemiology and for identifying candidate lagged covariates. SARIMA and related autoregressive models remain useful for capturing seasonal baselines and short-term autocorrelation in clinical use data. However, these models may be limited when high-dimensional covariates, complex nonlinear interactions, or nonstationary data-generating processes are present.

Recent deep learning methods provide additional options. Recurrent neural networks, long short-term memory networks, and attention-based architectures can model nonlinear temporal dependencies across multiple covariates. Temporal fusion transformers are especially relevant for multi-horizon forecasting because they can incorporate static variables, observed time-varying covariates, known future covariates, and attention-based interpretability mechanisms; recent biomedical forecasting studies have applied temporal fusion transformer–based models to clinical time-series prediction tasks [29]. Neural ordinary differential equation models and physics-informed neural networks provide another methodological direction by combining data-driven learning with continuous time dynamics or mechanistic constraints, which may be useful when the biological or epidemiological structure is partially known [30,31]. Online learning frameworks and adaptive state-space models are particularly relevant when the main challenge is not only nonlinear prediction but also continual updating under concept drift.

Therefore, this viewpoint does not argue that Kalman filtering or state-space models are universally superior to contemporary deep learning approaches. Rather, it emphasizes that future AECOPD early warning systems should be drift aware, updateable, and clinically interpretable. In practice, adaptive state-space methods, transformer-based models, neural differential equation models, and hybrid approaches may be complementary rather than mutually exclusive. The appropriate model class should be selected according to the target setting, data volume, interpretability requirements, update frequency, and the expected type of nonstationarity. To clarify the relative role of these approaches, Table 1 summarizes major forecasting model families that may be relevant to AECOPD early warning, with emphasis on their strengths, limitations, drift handling capacity, and relevance to multimodal surveillance.

**Table 1.** Forecasting model families for acute exacerbation of chronic obstructive pulmonary disease (AECOPD) early warning. This table provides a conceptual comparison of model families rather than a quantitative performance ranking. The relative strengths and limitations are summarized from the methodological literature discussed in the text.

Model family	Main strength	Key limitation	Drift handling	Relevance to AECOPD warning
DLNM^a^	Estimates nonlinear lagged exposure-response effects	Mainly explanatory; limited real-time updating	Periodic refitting	Identifies lagged temperature, pollution, and humidity effects
SARIMA^b^ and SARIMAX^c^	Captures seasonality and autocorrelation	Requires explicit handling of structural breaks and changing covariate effects	Rolling refitting or adaptive updating	Practical and interpretable backbone for forecasting seasonal AECOPD burden with environmental or behavioral covariates
GAM^d^ and regression time-series models	Interpretable nonlinear covariate adjustment	Limited under high-dimensional or drifting data	Dynamic terms or refitting	Supports environmental risk modeling and baseline adjustment
LSTM^e^ and RNN^f^	Models nonlinear multivariable temporal patterns	Data intensive; less interpretable	Retraining or online updating	Useful when multiple longitudinal signals are available
TFT^g^ and attention-based models	Supports multi-horizon forecasting and time-varying covariates	Requires large datasets and external validation	Monitoring and retraining	Promising for high-dimensional multimodal forecasting
Neural ODE^h^ and PINN^i^	Incorporates continuous time or mechanistic constraints	Complex; limited AECOPD evidence	Design dependent	Useful when the biological or epidemiological structure is known
Kalman and state-space models	Enables sequential updating and latent-state estimation	Requires state and noise assumptions	Strong online recalibration	Relevant for concept drift and changing care-seeking patterns
Hybrid and ensemble models	Combines complementary model strengths	Risk of overfitting and reduced transparency	Potentially strong with monitoring	Future direction for environmental, digital, and clinical fusion

a DLNM: distributed-lag nonlinear model.

b SARIMA: seasonal autoregressive integrated moving average.

c SARIMAX: SARIMA with exogenous variables.

d GAM: generalized additive model.

e LSTM: long short-term memory.

f RNN: recurrent neural network.

g TFT: temporal fusion transformer.

h ODE: ordinary differential equation.

i PINN: physics-informed neural network.

## Integration of Social Sensing Data

### Overview

While traditional environmental and meteorological data provide “etiological lead signals” (ie, the timing of physical or chemical stressors) for respiratory outbreaks such as AECOPD, a delay of several days to weeks typically occurs between extreme environmental exposure and formal medical consultation due to physiological tolerance and behavioral lag. To address this monitoring gap, social sensing data have been integrated into early warning frameworks as a high-frequency “behavioral lead signal.” Leveraging internet-based platforms and smart terminals, these heterogeneous data sources—including search engine logs and social media content—enable the real-time mapping of population-level health-seeking behaviors during the early phases of mild respiratory symptoms [32,33]. There is existing empirical evidence in digital epidemiology suggesting the early warning value of such unstructured data, particularly for acute infectious respiratory diseases. By using semantic mining and spatial clustering of health-related topics on social media, researchers successfully identified geographic signals of COVID-19 transmission across Europe during the winter of 2019 to 2020 prior to the official confirmation of the first indigenous cases by public health departments [34-36]. Similarly, by leveraging search query volumes from tools such as Google Trends, multiple studies have achieved accurate tracking of epidemic peaks for influenza and respiratory syncytial virus (RSV). These findings demonstrate that such data can serve as effective surrogate tools, particularly in settings where traditional clinical surveillance resources are limited [37-40]. In terms of algorithmic development, statistical ensemble frameworks, exemplified by augmented regression with grouped Google queries, have further demonstrated that incorporating internet search queries as exogenous covariates can significantly enhance the accuracy of time-series fitting for respiratory morbidity across multiple resolutions [41,42]. Given the digital media consumption patterns in mainland China, the Baidu index serves as a primary data source for localized digital surveillance [43,44]. Spatial epidemiological analyses indicate a strong and significant lagged correlation between the Baidu search index and the incidence of influenza-like illness across multiple regions in China. This provides a critical empirical foundation for the translation of such data into clinical early warning tools [45].

### AECOPD-Specific Evidence Gap in Digital Behavioral Surveillance

Despite the promise of digital behavioral surveillance, its transferability to AECOPD early warning remains uncertain. Most existing evidence supporting search query data, social media signals, and other internet-based behavioral indicators comes from acute infectious diseases, including COVID-19, influenza, and RSV. AECOPD differs from these conditions in several important respects. It occurs in a known chronic disease population; many patients have established rescue medications or written action plans; symptom deterioration may not immediately trigger internet searches; and health-seeking behavior is strongly influenced by baseline disease severity, access to care, and prior clinical experience. Therefore, direct empirical evidence for predicting AECOPD using internet search queries or social media signals remains limited. At this stage, digital behavioral signals should not be treated as validated predictors of AECOPD events but rather as candidate auxiliary signals that require disease-specific validation against clinical outcomes such as ED visits, hospital admissions, rescue medication use, or confirmed exacerbation episodes.

Accordingly, the role of social sensing in the conceptual framework discussed in this viewpoint should be interpreted cautiously. Digital behavioral data may help capture population-level attention, symptom concern, or care-seeking intention, but they should be integrated with environmental exposure data and clinical burden indicators rather than used as stand-alone early warning inputs. Future AECOPD-specific studies should evaluate whether search volumes, social media content, or platform-specific indexes provide incremental predictive value beyond meteorological variables, air pollution metrics, seasonal baselines, and recent clinical use data.

In summary, social sensing data should be viewed as a potentially useful but currently unvalidated auxiliary modality for AECOPD surveillance. Their main value may lie in complementing environmental exposure signals and clinical use data, particularly when evaluated within a disease-specific validation framework. Rather than assuming that findings from infectious disease surveillance can be directly transferred to AECOPD, future studies should quantify the incremental predictive value, temporal lead time, and population representativeness of digital behavioral indicators in patients with COPD.

### Equity, Digital Access, and Selection Bias

Even if digital behavioral signals prove useful for AECOPD surveillance, their use may introduce important equity and selection bias concerns. COPD burden is disproportionately concentrated among older adults, rural residents, and socioeconomically disadvantaged populations [2,3], yet these groups may have lower digital literacy; less consistent internet access; and lower engagement with search engines, social media platforms, or smartphone-based health apps. As a result, internet-based behavioral signals may overrepresent digitally active populations while underrepresenting the groups with the greatest AECOPD burden. This mismatch could lead to biased estimates of population risk, delayed detection in underserved communities, or early warning thresholds that perform unevenly across demographic and geographic subgroups.

Therefore, social sensing should not be used as a stand-alone surveillance layer for AECOPD. Instead, it should be integrated with more inclusive data streams, including meteorological and air pollution data, ED visits, hospital admissions, primary care records, medication use, community health management data, and local public health reporting. Future models should explicitly assess representativeness and fairness by stratifying performance across age groups, urban-rural residence, socioeconomic status, and baseline disease severity. Where feasible, subgroup-specific calibration, hierarchical modeling, or weighting strategies may be needed to prevent digital signals from amplifying existing inequities in respiratory health surveillance.

**Figure 1.**
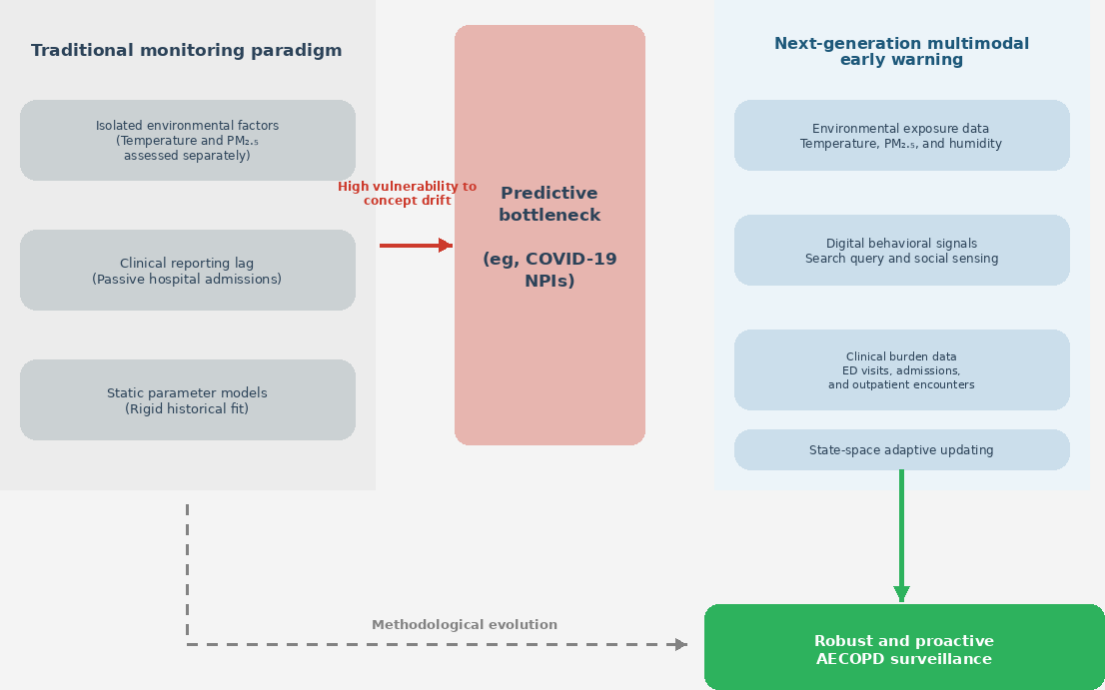
Conceptual framework of the paradigm shift in acute exacerbation of chronic obstructive pulmonary disease (AECOPD) early warning systems. The diagram illustrates the methodological evolution from the traditional monitoring paradigm, relying on isolated environmental factors, clinical reporting lag, and static models, to a next-generation multimodal early warning architecture. The revised architecture integrates environmental exposure data, digital behavioral signals, and clinical burden data as 3 input modalities, with state-space adaptive updating serving as an integration and drift-handling layer. The predictive bottleneck caused by extreme public health interventions such as COVID-19 nonpharmacological interventions (NPIs) highlights the need for drift-aware and proactive AECOPD surveillance. ED: emergency department.

Beyond the validity and representativeness of digital behavioral signals, another major challenge lies in the nonstationarity of multimodal time-series data. Although deep learning–based multimodal time-series warning models have demonstrated superior fitting accuracy under steady-state conditions, they are largely predicated on stationarity assumptions regarding historical data distribution. Consequently, the vulnerability of underlying algorithms is frequently exposed during sudden public health emergencies. During the 2020 COVID-19 pandemic, the global implementation of nonpharmacological interventions (NPIs) profoundly altered social activity trajectories. Leading epidemiological methodological studies have highlighted that the recurrent introduction of such exogenous interventions and drastic behavioral fluctuations directly resulted in significant performance degradation or total failure of numerous epidemiological prediction models during the pandemic [46]. Such macroscale physical disruptions and shifts in social behavior inevitably disrupted the traditional association pathways between meteorological or environmental exposure and non–COVID-19 respiratory diseases observed under previous steady states. Focusing further on clinical health care interfaces, empirical research has confirmed the impact of these structural breaks on algorithmic robustness. Taking ED load forecasting during the pandemic as an example, the COVID-19 outbreak induced severe “data drift” in patient health care–seeking characteristics and triage baselines. Due to drastic shifts in input feature distributions, machine learning models that previously performed optimally on steady-state historical data experienced a substantial decline in predictive accuracy [47]. Defined from the perspective of data science and statistical learning, the nonrandom shift in the mapping between input features and target variables induced by extreme disturbances such as a pandemic is formally termed “concept drift” [48]. Methodological research on health care load forecasting has explicitly asserted that traditional static early warning models with fixed parameters are highly susceptible to substantial biases when facing such systematic drift. Maintaining predictive accuracy necessitates the integration of algorithmic mechanisms capable of dynamic updating and the assimilation of new observations [48]. This underscores the necessity of moving beyond the limitations of static baselines when developing precise early warning systems for acute and chronic respiratory diseases such as AECOPD in the postpandemic era. These challenges highlight the need to explore adaptive model architectures capable of parameter updating and continuous assimilation of new observations in nonstationary AECOPD time-series data (Figure 1).

### Concept Drift Subtypes and Adaptive Responses in AECOPD Surveillance

Concept drift should not be treated as a single homogeneous phenomenon in AECOPD early warning. Different drift patterns may arise from different clinical, environmental, and social mechanisms, and each pattern may require a distinct adaptive response. Sudden drift may occur after abrupt external shocks, such as COVID-19 lockdowns, large-scale NPIs, hospital triage policy changes, or sudden disruptions in health care access. Gradual drift may emerge when treatment patterns, patient self-management behavior, or care-seeking preferences change progressively over time. Incremental drift may reflect long-term changes in population structure, disease management standards, air pollution profiles, or climate conditions. Recurring drift may arise from seasonal cold periods, haze episodes, holidays, or cyclical changes in respiratory disease burden.

Recognizing these subtypes is important because a single adaptive strategy is unlikely to address all nonstationary patterns. Sudden drift may require break point detection, intervention indicators, structural masking, or downweighting of affected periods. Gradual and incremental drift may be better handled through online learning, rolling recalibration, or time-varying coefficients. Recurring drift may require explicit seasonal state terms, periodic components, or calendar-aware model structures. Because concept drift in AECOPD surveillance may arise through different temporal patterns, adaptive responses should be matched to the specific drift subtype rather than applied uniformly (Table 2). Therefore, future AECOPD early warning systems should incorporate drift diagnosis before model updating rather than applying a uniform correction strategy to all forms of nonstationarity [46-48].

**Table 2.** Concept drift subtypes and candidate adaptive responses for acute exacerbation of chronic obstructive pulmonary disease (AECOPD) early warning.

Concept drift subtype	Plausible AECOPD surveillance example	Candidate adaptive response
Sudden	COVID-19 lockdowns, NPIs^a^, and hospital policy changes	Break point detection, masking or downweighting, and regime indicators
Gradual	Treatment pattern changes and changing care-seeking behavior	Online learning and rolling recalibration
Incremental	Population aging and long-term climate change	Time-varying coefficients and dynamic baseline updating
Recurring	Seasonal cold periods and recurrent haze episodes	Seasonal state terms and periodic components

a NPI: nonpharmacological intervention.

### A Minimal State-Space Formulation for Future AECOPD Forecasting

To make the proposed informatics perspective more explicit while avoiding overstatement, a minimal state-space formulation can be considered as a conceptual blueprint for future AECOPD forecasting rather than as a validated implementation. In such a framework, the latent-state vector could include several evolving components: underlying AECOPD burden; seasonal baseline risk; temperature response effects; air pollution response effects; a composite exposure-interaction component; digital behavioral signal contribution; and a drift component reflecting changes in population behavior, health care access, or clinical use patterns [47,48].

The observation layer could be defined using routinely collected clinical burden indicators such as daily or weekly AECOPD-related ED visits, hospital admissions, outpatient encounters, or exacerbation counts. Time-varying covariates may include meteorological variables, air pollution metrics, humidity, calendar effects, public health intervention indicators, and candidate digital behavioral signals. Rather than assuming fixed exposure-response relationships, these covariate effects could be allowed to evolve over time, enabling the model to update its estimates when environmental conditions, care-seeking behavior, or data-generating processes change [48].

Within this conceptual framework, sequential updating methods would revise latent-state estimates as new observations become available. A standard linear Kalman filter may be suitable when the system can be approximated as linear with Gaussian errors. Extended Kalman filters may be considered when the observation or transition equations are nonlinear, whereas unscented Kalman filters may be more appropriate when nonlinear exposure-response relationships are expected but local linearization may be unstable [49,50]. For higher-dimensional systems involving multiple interacting environmental, behavioral, and clinical data streams, ensemble Kalman approaches may provide additional flexibility, although their role in AECOPD surveillance remains to be empirically evaluated.

Importantly, this formulation is intended as a design consideration and research agenda, not as a fully specified or empirically validated forecasting system. Future AECOPD-specific studies should determine the optimal state representation, exposure interaction terms, update frequency, missing data strategy, and validation framework using real-world clinical surveillance data.

## Adaptive Correction and Regime-Aware Handling of Extreme Disturbances

### Overview

Public health emergencies, such as the COVID-19 pandemic, and associated response measures, such as NPIs, may trigger significant data and concept drift, rendering traditional time-series early warning algorithms—based on the assumption of “identically distributed historical data”—inadequate for postpandemic predictive needs. In the field of medical informatics, concept drift typically manifests as a systemic shift in the mapping between input features and predictive targets. Extreme social interventions such as physical distancing and mask wearing significantly altered both disease transmission pathways and population health care–seeking trajectories [47]. Empirical studies on ED triage and clinical prognosis have confirmed that such structural breaks in data cause static parameter models—including conventional machine learning and automated machine learning algorithms trained on steady-state historical data—to experience significant degradation in predictive performance or even complete failure [47,51]. On the basis of this methodological consensus, nonstationary social behavioral interference in environmental epidemiological forecasting similarly disrupts the static characterization of associations between “meteorological or environmental exposure” and “respiratory disease incidence,” significantly increasing the risk of bias in traditional early warning systems. To overcome these algorithmic robustness bottlenecks, developing multimodal intelligent early warning systems in the postpandemic era necessitates incorporating adaptive parameter correction mechanisms into the underlying architecture. Dynamic tracking algorithms based on state-space frameworks, including Kalman filtering and nonlinear variants such as the extended Kalman filter and unscented Kalman filter, have been explored for real-time epidemiological forecasting [49,50,52]. Rather than assuming fixed parameters, these approaches allow latent states or response parameters to evolve as new observations become available. In principle, this sequential updating structure may help recalibrate environmental or health care use baselines when social behavior, clinical access, or disease dynamics shift under extreme disturbances [49,50,52]. However, their value for AECOPD-specific surveillance remains to be empirically evaluated.

### Regime-Aware Handling of Structural Breaks

Extreme public health interventions such as lockdowns, mobility restrictions, mask mandates, or major changes in hospital access may introduce structural breaks into AECOPD surveillance time series. However, these periods should not automatically be treated as artifacts to be deleted. During COVID-19 lockdowns, reductions in AECOPD hospitalizations and mortality may partly reflect genuine changes in exposure, including reduced outdoor activity, decreased respiratory viral transmission, mask wearing, and altered air pollution or mobility patterns [53]. Therefore, structural masking should be understood as one option within a broader regime-aware modeling strategy rather than as a universal solution.

A cautious workflow should first identify potential break points using formal or semiformal procedures such as change point detection, cumulative sum monitoring, Bayesian change point methods, or interrupted time-series analysis [53,54]. Once a break point or intervention period is identified, the affected observations can be handled in several ways: retained with a regime indicator, downweighted during parameter estimation, flagged for sensitivity analysis, or masked only when the objective is to estimate counterfactual preintervention exposure-response relationships. In this sense, “masking” should not be equated with physical deletion of data.

Future AECOPD forecasting studies should report the proportion of observations affected by any masking or downweighting strategy because excessive exclusion may reduce effective sample size, weaken statistical power, and introduce selection or survivorship bias. Sensitivity analyses should compare alternative specifications, including no masking, downweighting, regime indicator adjustment, and counterfactual exclusion. If an intervention period contains a genuine epidemiological signal, such as a true reduction in exacerbation risk, it should be modeled as a distinct regime rather than removed from the learning process.

**Figure 2.**
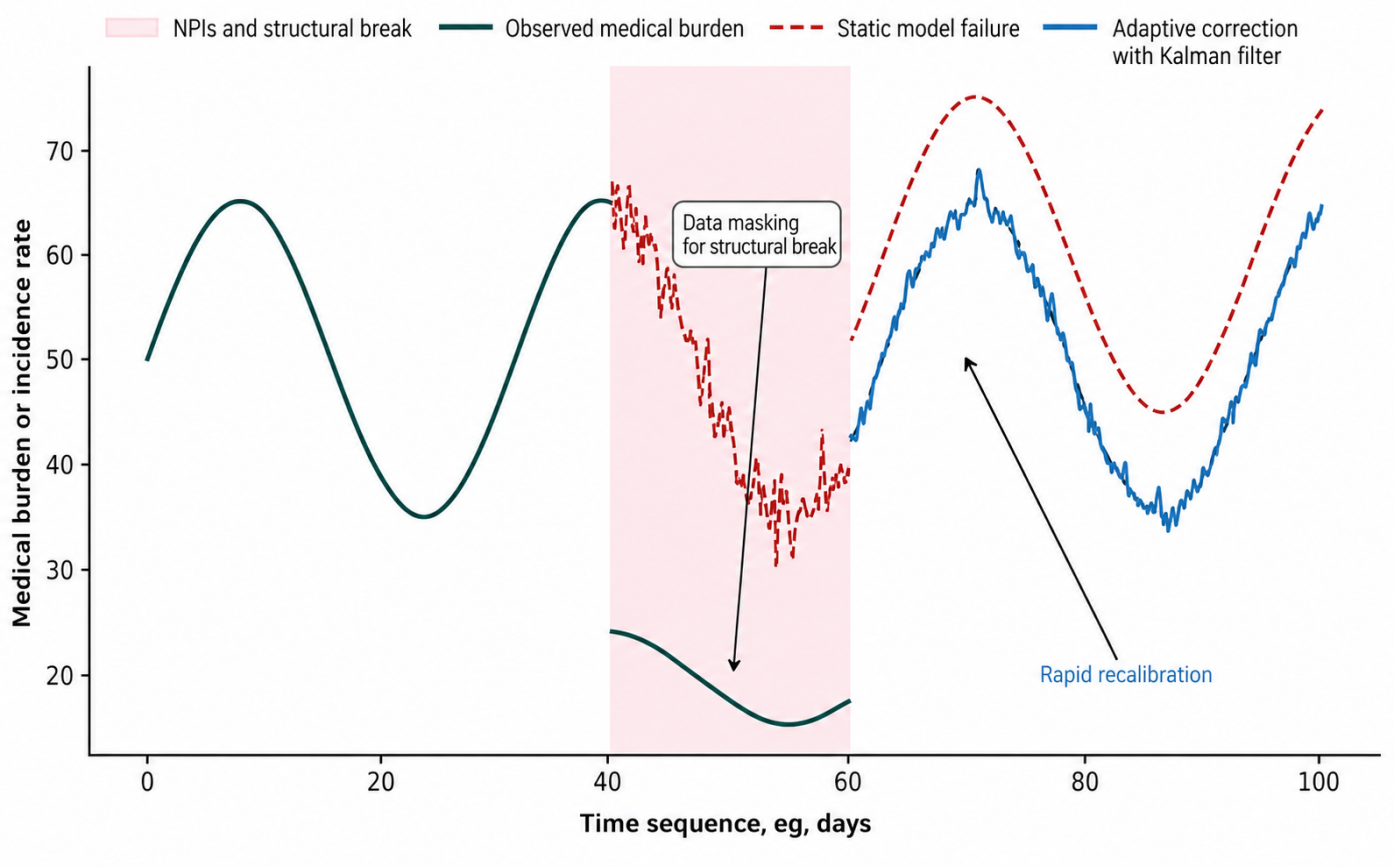
Illustration of concept drift and regime-aware adaptive correction in time-series forecasting under extreme exogenous interventions. The time-series plot illustrates how static parameter models may experience performance degradation when structural breaks occur, such as during nonpharmacological interventions (NPIs). Regime-aware strategies may include retaining observations with intervention indicators, downweighting affected periods, conducting sensitivity analyses, or applying masking only for specific counterfactual objectives. State-space adaptive filtering can then support sequential recalibration after the intervention period. This figure is conceptual and does not represent a validated acute exacerbation of chronic obstructive pulmonary disease (AECOPD) forecasting model.

In summary, state-space adaptive correction and regime-aware handling of structural breaks may improve the robustness of future AECOPD early warning models under nonstationary conditions. However, masking should be used selectively and justified according to the analytic objective. In some settings, retaining intervention periods with regime indicators or downweighting affected observations may be preferable to excluding them. Future studies should compare these strategies empirically before recommending any single approach as standard practice. More broadly, drift-aware adaptive updating may help recalibrate predictive baselines when public health emergencies alter clinical use patterns [47,51], but its value for AECOPD surveillance should be evaluated in real-world datasets before being translated into operational early warning systems (Figure 2).

## Limitations

Several limitations should be acknowledged. First, this viewpoint is not a systematic review, meta-analysis, or formal methodological validation study. The evidence base was drawn from representative literature in environmental epidemiology, respiratory medicine, digital epidemiology, and medical informatics and was used to develop a conceptual research agenda rather than an exhaustive synthesis of all available evidence. Therefore, the selection and interpretation of studies may be influenced by the inherent subjectivity of narrative synthesis. Gray literature was not systematically assessed, and the discussion should be interpreted as hypothesis generating rather than definitive.

Second, the proposed framework has not yet been empirically validated in an AECOPD-specific surveillance dataset. Direct evidence supporting internet search queries, social media signals, or other digital behavioral indicators for AECOPD prediction remains limited, and findings from COVID-19, influenza, or RSV surveillance cannot be assumed to transfer directly to COPD exacerbation forecasting. In addition, social sensing data may underrepresent older adults, rural residents, and socioeconomically disadvantaged populations, who often bear a high burden of COPD. Finally, several implementation questions remain unresolved, including the optimal state-space formulation, update frequency, missing data handling, breakpoint detection strategy, threshold calibration, external validation requirements, and integration with real-world clinical workflows.

## Conclusions and Future Perspectives

This viewpoint argues that AECOPD early warning should move beyond static exposure-response modeling toward drift-aware, multimodal, and equity-conscious surveillance. The proposed direction is not a validated forecasting system but a research agenda for integrating environmental exposure data, candidate digital behavioral signals, and routinely collected clinical burden indicators under nonstationary conditions. Future studies should validate whether digital behavioral signals add incremental predictive value for AECOPD; compare static and adaptive forecasting models in real-world clinical datasets; evaluate drift subtype–specific updating strategies; and report performance across age, urban-rural residence, socioeconomic status, and baseline disease severity. Implementation will also require transparent data infrastructure, predefined handling of structural breaks, sensitivity analyses for masking or regime adjustment, and clinically interpretable warning thresholds. These steps are necessary before drift-aware multimodal early warning can be responsibly translated into AECOPD prevention and health care resource planning.

## Abbreviations

AECOPD: acute exacerbations of chronic obstructive pulmonary disease
COPD: chronic obstructive pulmonary disease
DLNM: distributed-lag nonlinear model
ED: emergency department
NPI: nonpharmacological intervention
RSV: respiratory syncytial virus
SARIMA: seasonal autoregressive integrated moving average
